# Stability and Antimicrobial Efficacy of Reuterin and Bacteriocins (Microcin J25, Nisin Z, and Pediocin PA-1) in Chitosan- and Carboxymethyl-Cellulose-Based Hydrogels

**DOI:** 10.3390/microorganisms13102249

**Published:** 2025-09-25

**Authors:** Samira Soltani, Muriel Subirade, Eric Biron, Christophe Cordella, Gabriel Romondetto, Ismail Fliss

**Affiliations:** 1 Canadian Research Institute for Food Safety, Department of Food Science, University of Guelph, Guelph, ON N1G 2W1, Canada; 2 Food Science Department, Food and Agriculture Faculty, Laval University, Quebec, QC G1V 0A6, Canada; muriel.subirade.1@ulaval.ca (M.S.); christophe.cordella@fsaa.ulaval.ca (C.C.); ismail.fliss@fsaa.ulaval.ca (I.F.); 3 Institute of Nutrition and Functional Foods, Laval University, Quebec, QC G1V 0A6, Canada; eric.biron@pha.ulaval.ca; 4 Faculty of Pharmacy, Laval University and Laboratory of Medicinal Chemistry, CHU de Québec Research Center, Quebec, QC G1V 4G2, Canada; 5 LARTIC, Laboratoire de Recherche et de Traitement de l’Information, Chimiosensorielle Laval University, Quebec, QC G1V 0A6, Canada; 6 Coopérative Agropur, 4700 Rue Armand Frappier, Saint-Hubert, QC J3Z 1G5, Canada; gabriel.remondetto@agropur.com

**Keywords:** bacteriocins, reuterin, chitosan, CMC, sanitizers, hydrogel

## Abstract

Traditional chemical-based sanitizers pose risks to health and the environment, highlighting the need for safer natural alternatives. We developed biocompatible hydrogels from carbohydrate-based biopolymers, chitosan (1.5% and 2.5%), and carboxymethylcellulose (CMC, 3% and 5%), each incorporating one of four antimicrobials: microcin J25, nisin Z, pediocin PA-1, or reuterin. Hydrogels were prepared by dissolving the polymers in aqueous solution and incorporating antimicrobials before gelation. The formulations were characterized using viscosity measurements, antimicrobial assays, and stability testing over 28 days of storage at room temperature (23–25 °C). Chitosan hydrogels with microcin J25 maintained strong activity against *Salmonella* enterica ATCC 6962, while nisin Z retained activity in gel and solution forms, though with some decline during storage. Pediocin PA-1 remained active in 1.5% and 2.5% chitosan hydrogels against *Listeria monocytogenes* ATCC 19115, but activity was lost in 3% and 5% CMC hydrogels. Reuterin preserved activity in CMC-based hydrogels throughout storage. In solution, microcin J25 and nisin Z consistently achieved ~7-log reductions, whereas pediocin PA-1 and reuterin reached up to ~5-log reductions. In gels, efficacy decreased at lower concentrations and shorter contact times, likely due to diffusion barriers. Overall, the hydrogels remained stable during storage, and CMC- and chitosan-based matrices with selected antimicrobials show promise as alternatives to chemical sanitizers. Their application should be tailored to specific needs, with formulations requiring longer contact times best suited for surfaces that allow prolonged exposure.

## 1. Introduction

Foodborne diseases pose a significant threat to public health, with over 48 million cases reported annually in the United States and more than 600 million globally [1,2]. Contamination through infected food handlers, who fail to properly wash or sanitize their hands and surfaces, remains one of the leading contributors to foodborne outbreaks [3]. Proper sanitization of work surfaces and hands is a critical factor in minimizing contamination risks, not only in the food service industry but also in healthcare, veterinary practices, and other environments where hygiene is crucial. In food services, hand sanitizers are a viable alternative to hand washing with soap, provided hands are not visibly soiled [4].

Similarly, in veterinary clinics and healthcare settings, effective surface sanitization and hand hygiene are essential to prevent the transmission of zoonotic and nosocomial infections [5,6]. These broader applications highlight the universal importance of developing efficient, safe, and environmentally friendly sanitizing solutions. Hand sanitizers are mainly categorized as alcohol-based hand sanitizers (ABHSs) and non-alcohol-based hand sanitizers (NABHSs), and they are commercially available as liquid, gels, rubs, wipes, etc. The active ingredient in an ABHS comprises one or more type of alcohol, including ethanol, isopropanol, and *n*-propanol at a concentration of 60–95%. However, the active ingredients in a NABHS comprise chemicals with antiseptic properties, such as quaternary ammonium compounds, triclosan, chlorohexidine, and iodine/iodophors, etc. [7]. Owing to their low cost and effectiveness, ABHS are routinely incorporated as a crucial part of infection control protocols in healthcare facilities [8]. Nevertheless, there are several safety concerns associated with ABHS, such as flammability and potential toxicity when accidentally ingested [9,10]. Additionally, alcohol or hydrogen peroxide in these sanitizers can cause eye and skin irritation. Hence, their excessive use can cause skin damage and irritation and peeling with redness or itching, making the skin highly susceptible to microbial contamination [11]. Moreover the frequent use of alcohol, hydrogen peroxide, and surfactants causes resistance in microorganisms [12]. Notably, certain pathogens have developed resistance against alcohol because of the broad spectrum of inhibitory action of alcohols; this has raised concerns regarding the efficacy of hand sanitizers [13,14]. Furthermore, alcohol spill in a water body is toxic to aquatic organisms [11]. Studies have also documented the contribution of triclosan and quaternary ammonium compounds in environmental toxicity and bacterial resistance [8,15]. Importantly, chemical surfactants are being considered as an emerging contaminant, because they are not easily degradable and remain in the environment for a long time, thus contributing to environmental pollution [9].

Hence, there has been a long-standing demand for sanitizers or antimicrobial hydrogels that have a high degree of antimicrobial efficacy, with environmental compatibility. Importantly, to minimize the risk of dermal or oral toxicity upon accidental exposure, the active compounds used in such formulations should be carefully selected and evaluated for safety. While natural antimicrobials are often favored for their biocompatibility and consumer acceptance, natural origin does not guarantee non-toxicity. Indeed, certain natural products, such as ricin, aflatoxins, tetrodotoxin, coniine, and cyanogenic glycosides, are highly toxic. Therefore, each compound must undergo rigorous safety assessments before application.

Natural antimicrobial compounds, such as bacteriocins, organic acids, and reuterin, which are derived from bacteria has attracted substantial attention in recent decades. Due to their broad or narrow spectrum of activity, high potency, and low toxicity, they have been considered as a potential alternative candidate and ideal active ingredients in sanitizers for application in different sectors [16,17]. Bacteriocins are antimicrobial peptides synthesized by ribosomes and produced by both Gram-positive and Gram-negative bacteria. They are categorized into Class I and Class II based on the presence or absence of posttranslational modifications. Their strong antimicrobial activity at low concentrations, along with heat stability and resistance to extreme pH and salt conditions, make bacteriocins effective against closely related bacteria. With a low risk of resistance development, they hold great potential for applications in food preservation, veterinary medicine, and healthcare, attracting growing interest in research and industry [18]. Several studies have demonstrated that bacteriocins are potent against bacteria that are relevant in clinical settings and in foods [19,20]. Microcin J25 is a Class II lasso peptide produced by certain strains of *Escherichia coli*. It is characterized by its unique lasso structure that confers exceptional stability to heat and proteolysis. Its primary mechanism of action involves the inhibition of RNA polymerase and interference with membrane potential, making it highly effective against a range of Enterobacteriaceae [21]. Nisin Z, produced by *Lactococcus lactis*, is a Class I lantibiotic containing lanthionine rings that enable pore formation in bacterial membranes, leading to rapid cell death. It is generally recognized as safe (GRAS) and has extensive applications in food preservation and biomedical formulations [22]. Pediocin PA-1, a Class IIa bacteriocin produced by *Pediococcus acidilactici,* exhibits strong activity against *Listeria monocytogenes* by targeting the mannose phosphotransferase system on the cell membrane [23]. These bacteriocins have attracted attention for advanced applications in formulation science, particularly in food coatings, disinfectant systems, and other biocompatible delivery matrices [24,25].

Reuterin is another natural antimicrobial produced by *Limosilactobacillus reuterin* via the bioconversion of glycerol. It has a broad spectrum of activities and targets fungi and Gram-positive and Gram-negative bacteria [26]. Both bacteriocin and reuterin are water-soluble and stable at a wide range of pH and salt concentrations; thus, these compounds can potentially be used in sanitizers. However, very few studies have considered their potential application as sanitizers.

Hydrogel formulations have multiple advantages over liquid formulations because they are more stable, easy to handle and apply, are more spreadable, and can penetrate into organisms [27]. Hence, sanitizer hydrogel formulations are becoming increasingly popular in the market. Incorporating bacteriocins into polymer-based hydrogels presents challenges related to stability, bioavailability, and release. Interactions Interactions with hydrogel components may reduce their bioavailability, limiting diffusion and antimicrobial activity Additionally, hydrogels often result in slower release, which can weaken immediate efficacy [28,29]. Optimizing hydrogel composition is crucial to maintaining bacteriocin activity for effective applications in sanitation and food safety.

Previously, using different in vitro assays, we showed that bacteriocins and reuterin are not toxic at their effective concentrations (MIC, MBC). Specifically, they did not cause skin irritation or sensitization [17]. In this study, we evaluated the behavior, stability, bioavailability, and antimicrobial activity of different classes of bacteriocins in various natural biopolymers. The ultimate goal was to develop biocompatible hydrogels with antimicrobial properties targeting the most problematic pathogenic and spoilage microorganisms in the food and medical sectors. In this regard, we produced and purified four natural antimicrobial compounds: microcin J25, nisin Z, pediocin PA-1 and reuterin. Each antimicrobial compound was incorporated into the hydrogel formulations that were developed from the carbohydrate-based biopolymers chitosan and CMC. Each biopolymer was prepared using two formulations (3% and 5% CMC and 1.5%, and 2.5% chitosan) and had two viscosities. Eventually, we characterized the developed hydrogels based on their general characteristics, viscosity, antimicrobial activity, and stability when stored at room temperature.

## 2. Materials and Methods

### 2.1. Chemicals

Low-molecular-weight chitosan (deacetylated chitin, poly(D-glucosamine); Sigma-Aldrich, Product No. 448869, St. Louis, MO, USA; CAS: 9012-76-4) with a degree of deacetylation > 75% was used for hydrogel preparation. Sodium carboxymethyl cellulose (CMC; MW 90,000; Sigma-Aldrich; CAS: 9004-32-4) was used as an alternative polymer. Calcium chloride (CaCl_2_; Sigma-Aldrich; CAS: 10043-52-4), lactic acid (≥90%; Sigma-Aldrich; CAS: 50-21-5), hydrochloric acid (HCl; Fisher Scientific, Ottawa, ON, Canada; CAS: 7647-01-0), acetonitrile (HPLC grade; Fisher Scientific, 4 L; CAS: 75-05-8), potassium phosphate monobasic (KH_2_PO_4_; Sigma-Aldrich; CAS: 7778-77-0), and glycerol (Sigma-Aldrich; CAS: 56-81-5) were also obtained. All other chemicals used were of analytical grade. Distilled and deionized water were used throughout the experiments. All the other chemicals used in this study were of analytical grade. Distilled and deionized water were used throughout the experiment.

### 2.2. Bacterial Strains and Growth Conditions

*Escherichia coli* MC4100 PTUC 202 (STELA Collection, Laval University, Quebec City), used for MccJ25 production, was cultured aerobically at 37 °C overnight in Luria-Bertani (LB) (Difco Laboratories, Spark, MD, USA). *Limosilactobacillus reuteri* ATCC 53608, used for reuterin production, was cultured at 37 °C overnight in Man–Rugosa–Sharpe (MRS) (Oxoid, Nepean, ON, Canada), under anaerobic conditions. *Staphylococcus aureus* ATCC 6538 and *Listeria monocytogenes* ATCC 19115 were used as Gram-positive indicator strains and *Salmonella enterica* subsp. *enterica* serovar Newport ATCC 6962 (later referred to as *S*. Newport, ATCC 6962) (STELA Collection, Laval University, QC, Canada) was used as the Gram-negative indicator strain. *S. aureus* ATCC 6538 was grown on Tryptic soy broth (Difco Laboratories, Sparks, MD, USA), and *L. monocytogenes* ATCC 19115 was grown on Brain Heart Infusion (Difco Laboratories, Sparks, MD, USA) and incubated at 37 °C. *S.* Newport ATCC 6569 were grown on LB broth and incubated at 37 °C. Working cultures were maintained on agar plates, TSB, BHI and LB agar, all supplemented with 15 g/L agar (Thermo Fisher Scientific, Waltham, MA, USA) prior to the experiment. Broth cultures were grown directly from an individual colony of thestrain in respective media and incubated overnight at appropriate concentration as described above. All cultures were stored frozen (−80 °C) in respective media supplemented with 20% (*v*/*v*) sterile glycerol.

### 2.3. Production and Purification of Compounds

*Microcin J25* (MccJ25) was produced from the culture supernatant of *E. coli* MC400 PTUC202 cultured in minimal medium (M63) according to [16]. Briefly, MccJ25 was pre-purified from the culture supernatant via solid-phase extraction using a Sep-Pak C18 35 cc Cartridge (Water) and eluted using acetonitrile/water (30% *v*/*v*) containing 0.1% HCl and further purified to homogeneity (up to 95% purity) via RP-HPLC (Beckman Coulter System Gold Preparative HPLC system, Mississauga, ON, Canada) on a preparative C18 column (Luna 10 µm, 250 mm × 21.10 mm, Phenomenex, CA, USA). The purified sample (microcin J25) was quantified using an RP-HPLC system (Waters, Milford, MA, USA) equipped with an analytical C18 column.

*Nisin Z* Nisin Z was obtained from a commercial preparation (Niseen-S, Fromagex, Rimouski, QC, Canada). The powder was dissolved in Milli-Q water and subjected to solid-phase extraction using a Sep-Pak C18 35 cc Cartridge (Waters Corporation, Milford, MA, USA) pre-equilibrated with 0.1% trifluoroacetic acid (TFA). Bound peptides were eluted with 30% (*v*/*v*) acetonitrile in water containing 0.1% TFA at a flow rate of 2.5 mL/min. The fractions containing nisin Z were pooled, concentrated using a Speed-Vac overnight at 45 °C, and lyophilized. The purified sample was quantified, and purity was confirmed via RP-HPLC (Waters, Milford, MA, USA) equipped with an analytical C18 column (Aeris 3.6 μm PEPTIDE XB-C18, 250 × 4.6 mm, Phenomenex, CA, USA).

*Pediocin PA-1* was synthesized via Fmoc-based solid-phase peptide synthesis (SPPS) on a Rink amide resin (Novabiochem) as previously described by Bédard et al. (2018) [23], with minor modifications. Amino acids were coupled using HBTU/HOBt activation, and the peptide was cleaved from the resin with trifluoroacetic acid (TFA) containing standard scavengers. The crude peptide was precipitated in cold diethyl ether, dissolved in water, and purified to >95% homogeneity by RP-HPLC on a preparative C18 column (Phenomenex Luna, 250 × 21.1 mm). Purity was confirmed via analytical RP-HPLC using an Aeris 3.6 μm PEPTIDE XB-C18 column (250 × 4.6 mm, Phenomenex, CA, USA).

*Reuterin* was obtained from the bioconversion of glycerol by *Liml. reuteri* as per the conditions previously described [30]. The *Liml. reuterin* culture was grown anaerobically in MRS media supplemented with 20 mM glycerol at 37 °C overnight. Following incubation, the culture was centrifuged (1500× *g*, 10 min, 20 °C) and the cells were washed twice (with 0.1 M potassium phosphate) and resuspended in 300 mM glycerol and incubated anaerobically at room temperature for 45 min. Bacterial suspensions were centrifuged, and the supernatant was filtered. The purity and quantity of reuterin were determined using an analytical HPLC system (Waters, Milford, MA, USA) equipped with ICsep-ion-300 column (Transgenomic, San Jose, CA, USA).

### 2.4. Hydrogel Preparation

*CMC hydrogels:* Carboxymethyl cellulose (CMC) was dissolved at 3% and 5% (*w*/*v*) in distilled water under constant magnetic stirring (500 rpm) at room temperature (~22–23 °C) for 12 h to ensure complete dissolution. Once fully dissolved, antimicrobial agents were added directly to the polymer solution and mixed for 15 min. Calcium chloride (CaCl_2_) was then added dropwise to reach a final concentration of 1.5% (*v*/*v*), and the mixture was stirred for 30 min at 400 rpm to induce crosslinking and hydrogel formation.

*Chitosan hydrogels*: Chitosan was dissolved at 1.5% and 2.5% (*w*/*v*) in distilled water containing the antimicrobial agents. Lactic acid (1% *v*/*v* final concentration) was added dropwise under continuous stirring (500 rpm) at room temperature until a pale-yellow hydrogel was formed. Stirring continued for 30 min to ensure homogeneity.

The selected concentrations (1.5% and 2.5% chitosan; 3% and 5% CMC) were chosen to represent two viscosity ranges: one more fluid-like for surface applications and one more viscous for hand-sanitizer-type use, based on the preliminary screening of polymer performance.

### 2.5. Incorporation of Antimicrobial Agents and Stability Analysis

To evaluate stability over time, antimicrobial agents were incorporated into the hydrogels at concentrations corresponding to 50 times their minimum inhibitory concentrations (MICs), which had been previously determined against the selected indicator strains (*S*. Newport ATCC 6962, *S. aureus* ATCC 6538, and *L. monocytogenes* ATCC 19115). The concentrations used were 1.78 µg/mL for microcin J25, 75 µg/mL for nisin Z, 4.5 µg/mL for pediocin PA-1, and 5 mg/mL for reuterin. These hydrogels were analyzed for flow behavior, pH stability, and antibacterial activity over four weeks at room temperature to evaluate storage stability.

### 2.6. Efficacy Testing at Higher Antimicrobial Concentrations

To assess antibacterial efficacy, hydrogels were prepared with higher concentrations of antimicrobial agents. The concentrations tested included microcin J25 at 250 µg/mL, 500 µg/mL, and 1000 µg/mL; nisin Z at 250 µg/mL, 500 µg/mL, and 1000 µg/mL; pediocin PA-1 at 250 µg/mL, 500 µg/mL, and 1000 µg/mL; and reuterin at 250 mg/mL, 500 mg/mL, and 1000 mg/mL. Hydrogels formulated with these concentrations were evaluated for log reduction efficacy following the AOAC 960.09 Germicidal and Detergent Sanitizing Action of Disinfectants protocol.

### 2.7. Morphology of Hydrogels

The morphology of CMC (3%) and chitosan (1.5%) were assessed using TEM (JEM-1230; JEOL Ltd., Tokyo, Japan) equipped with a Gatan UltraScan 1000XP camera (Gatan Inc., Pleasanton, CA, USA). Samples were dehydrated via standard preparation prior to imaging, which may lead to the partial collapse or alteration of the hydrated gel network. Conventional TEM often involves dehydration steps that can collapse the native hydrogel network, limiting accurate microarchitecture visualization [31]. Advanced techniques such as cryo-SEM, ESEM, or freeze-substitution are better suited to preserving hydrated structures and avoid artifacts, as demonstrated in recent comparative studies.

### 2.8. Flow Behavior and Viscosity Measurements

The viscosity of hydrogels was measured using ARES-100 FRT (Rheometric Scientific. Piscataway, NJ, USA), equipped with a cylinder measuring system (33.93 mm cup diameter, 32.05 mm bob diameter, 33.29 mm bob length). Analyses were carried out at room temperature (20 ± 2 °C); approximately 25 mL of each sample was poured into a temperature-controlled measuring vessel and subjected to shear rates ranging from 0.01 to 100 s^−1^ with 3 s intervals. The relationship between shear stress (σ) and shear rate (

$$\dot{\gamma }$$)

was fitted to the Herschel–Bulkley model [32,33]:

$${\sigma \;=\;\sigma_{0}+\;K\dot{\gamma }}^{n}$$

where σ_0_ is the yield stress (mPa), K is the consistency index (mPa·s), and n is the flow behavior index. Fitting parameters were obtained by nonlinear regression, and model quality was assessed by reporting the coefficient of determination (R^2^) and standard errors of the parameter estimates.

### 2.9. pH Evaluation

The pH measurement of the different hydrogel formulations was carried out using a digital pH meter (VWR, International, Radnor, PA, USA), equipped with a Thermo Scientific™ Orion™ flat-surface combination electrode (model 913600, Thermo Fisher Scientific, Waltham, MA, USA) designed for semi-solid samples. Measurements were performed in triplicate, and results are expressed as the mean ± SD.

### 2.10. Antimicrobial Activity Test

Agar well diffusion assay: The antibacterial activity of developed hydrogels were assessed qualitatively via an agar well diffusion assay, as previously described by [30]. Briefly, indicator strains were cultured as per the conditions previously described. TSB and LB agar plates (0.75% *w*/*v* agar) were seeded with the overnight culture (at 1% concentration) of indicator strains. Wells were created on agar plates using sterile glass pipettes, and then approximately 80 µL of hydrogel was added to wells. Following an overnight incubation of the plates in appropriate conditions, the zone of inhibitions was recorded. Pictures of the plates were taken with ChemiDoc XRS (Bio-Rad, Hercules, CA, USA).

Minimum inhibitory concentration assay (MIC): MICs of developed hydrogels were determined as previously described by [34]. Briefly, a flat-bottom sterile 96-well plate was filled with 125 µL of respective media and 125 µL of hydrogels (diluted 2 times) were added and two-fold serial dilution was performed along the row. An overnight culture of indicator strains at the concentration of 10^5^ CFU/mL was added to each well (50 µL). In case of *S.* Newport ATCC 6962, an overnight culture was sub-cultured in fresh LB broth and allowed to grow to an OD_595_ of 1, before adjusting to the final concentration of 10^5^ CFU/mL. MIC assays were performed using three biological replicates. Plates were incubated overnight under appropriate conditions before the OD_595_ was measured. MIC values were determined in three biological replicates and correspond to the lowest concentration exhibiting significant inhibition of the sensitive strain.

### 2.11. Evaluation of Antimicrobial Efficacy

The efficacy of the formulated hydrogels and bacteriocins was assessed using the AOAC 960.09 protocol for Germicidal and Detergent Sanitizing Action of Disinfectants, with slight modifications [35]. Formulations in solution and gel forms were prepared at the desired concentrations, and 9.9 mL of each was mixed with 100 µL of a bacterial suspension to achieve an initial bacterial concentration of approximately 10^7^–10^8^ CFU/mL. The mixture was incubated at 25 ± 1 °C for specific contact times (30 s, 1, 5, and 30 min). After the designated contact time, 1 mL of the treated sample was transferred to 9 mL of neutralizer solution (corresponding to a 10^−1^ dilution tube) to stop the activity of the sanitizer.

The neutralizer solution was prepared by mixing 40 g lecithin and 280 mL polysorbate 80 with 1.25 mL of 0.25 M phosphate buffer and diluting to 1 L with sterile water. The solution was sterilized prior to use. Neutralized samples were serially diluted in sterile phosphate-buffered dilution water (PBDW) to a final dilution of 10^−7^, and aliquots were plated onto appropriate agar media. Plates were incubated at 36 ± 1 °C for 24–30 h, and colony-forming units (CFUs) were enumerated to determine the surviving bacterial population. Log reduction was calculated by comparing the logarithmic values of the initial bacterial population to those of the treated samples.

Neutralizer efficacy was confirmed in accordance with AOAC guidelines to ensure it effectively halted the activity of the sanitizers without affecting bacterial viability. Validation tests included assessing the ability of neutralizer to inactivate the sanitizer within 30 s and confirm it was non-toxic to the test organism. The difference between bacterial counts in treated and control samples did not exceed 1 log, validating the performance of the neutralizer.

### 2.12. Statistical Analysis

Statistical analyses were performed using one-way analysis of variance (ANOVA) (*p* < 0.0001) with Tukey’s multiple comparisons test using GraphPad Prism macOS version 10.4.0 (GraphPad Software, San Diego, CA, USA) and shown as mean  ±  standard error of mean (SEM). Principal component analysis (PCA) and partial least-square modeling (PLS) were used to study the correlations among hydrogels using the SAISIR Package and MATLAB (version R2021b, MathWorks Inc, Natick, MA, USA) [36]. The results for viscosity and pH measurement are presented as the mean and SD of at least three replicates.

## 3. Result

### 3.1. Production and Purification of Antimicrobial Compounds

**Figure 1:**
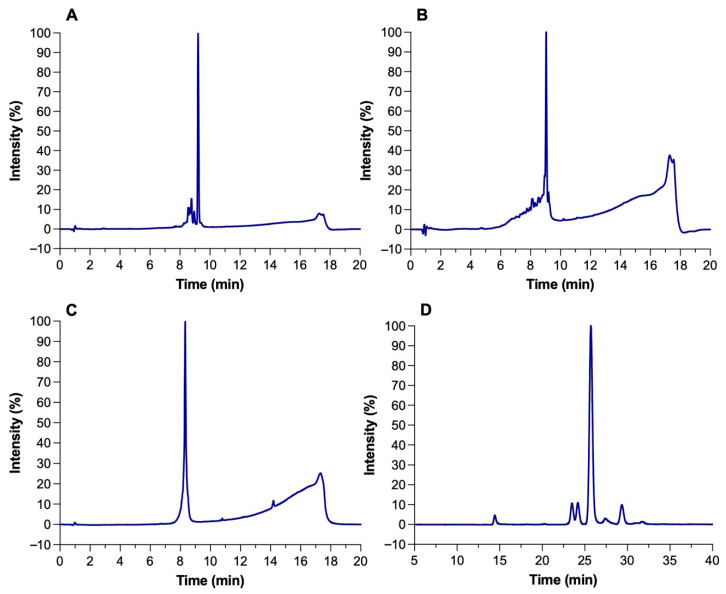
HPLC chromatograms of (**A**) microcin J25, (**B**) nisin Z, (**C**) pediocin PA-1, and (**D**) reuterin.

The identity and purity of microcin J25 (MccJ25), nisin Z, pediocin PA-1, and reuterin were confirmed by analytical RP-HPLC. Each compound exhibited a single dominant peak at its expected retention time with minimal impurities (Figure 1A–D). Reported retention times were consistent with published values for these compounds under similar RP-HPLC conditions [17]. Peak purity analysis confirmed that the main peaks represented >90% of total area, with nisin Z and pediocin PA-1 exceeding 95%. Reuterin, obtained through the bioconversion of 300 mM glycerol by *Liml. reuteri* ATCC 53608, reached a final concentration of ~200 mM with an 85% yield. The HPLC profile showed no residual glycerol, indicating efficient conversion and high product purity (Figure 1D).

### 3.2. Hydrogel Formulations

**Figure 2:**
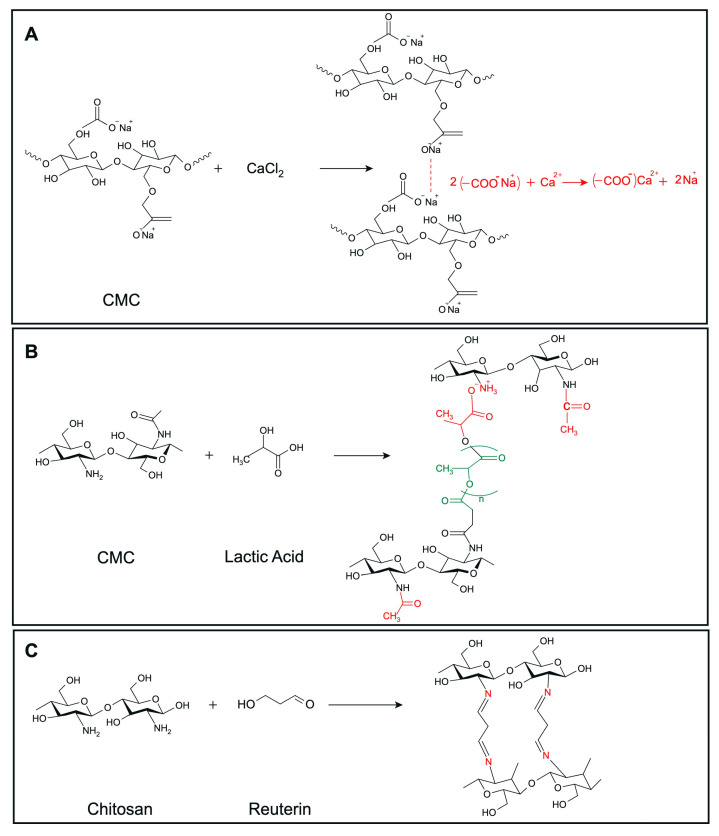
Predicted crosslinking reactions of (**A**) CMC with CaCl2, (**B**) chitosan with lactic acid, (**C**) chitosan with reuterin. Structures were drawn using MarvinSketch, Web version (ChemAxon Ltd., Budapest, Hungary).

We developed a range of hydrogels based on CMC (3% and 5% *w*/*v*) and chitosan (1.5% and 2.5% *w*/*v*), incorporating various antimicrobial agents, including microcin J25, nisin Z, pediocin PA-1, and reuterin. The crosslinking reaction between CMC and calcium chloride is depicted in Figure 2A. The addition of microcin J25, nisin Z, pediocin PA-1, and reuterin to CMC hydrogels did not visibly alter their color or structure. The mechanism underlying the formulation and lactic-acid-based crosslinking of chitosan is depicted in Figure 2B. Similarly, the incorporation of microcin J25, nisin Z and pediocin PA-1 into chitosan hydrogels did not change their physical properties, such as color. However, the addition of reuterin to chitosan hydrogels formed a solid gel with a distinct red color, likely due to a reaction between the carbonyl group of reuterin and the amine group of chitosan (Figure 2C). As a result, this formulation was excluded from further analysis.

For efficacy testing, hydrogels were prepared with different concentrations of antimicrobial agents, including microcin J25 (250, 500, 1000 µg/mL), nisin Z (250, 500, 1000 µg/mL), pediocin PA-1 (250, 500, 1000 µg/mL), and reuterin (250–1000 mg/mL). Their bactericidal efficacy of the different hydrogels was assessed through the determination of bacterial log reduction. No significant visual changes in the physical appearance of hydrogels were observed at these concentrations.

### 3.3. Morphology of Hydrogels

**Figure 3:**
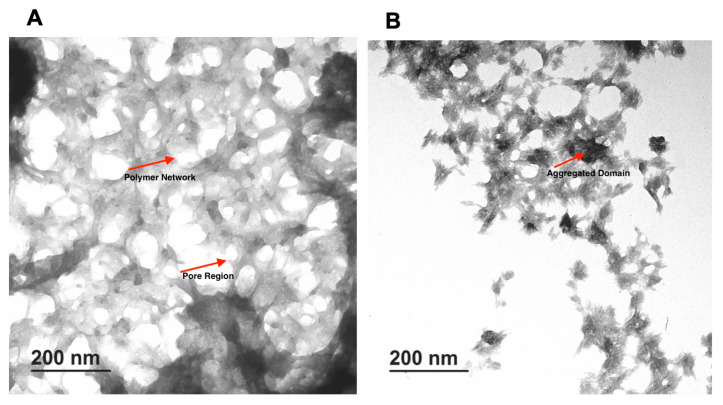
TEM images of formulated hydrogels. (**A**) CMC 3%, (**B**) Chitosan 1.5%.

The TEM image of CMC 3% and chitosan 1.5% are shown in Figure 3. A uniform porous structure for both types of gels was observed. Pores in both hydrogels have a mostly circular ellipse shape with different sizes and are well connected to each other. The porous structure seems to depend on the final concentration of the polymer. The CMC 3% hydrogel appeared more compact, with fewer pores compared to the chitosan 1.5% hydrogel. The impact of polymer concentration on final hydrogel structure was also evident with the two different concentrations of the same polymer. In particular, the hydrogel made from 3% CMC was more porous than 5%, and a similar result was observed for 1.5% and 2.5% chitosan.

### 3.4. Viscosity and Flow Behavior

**Figure 4:**
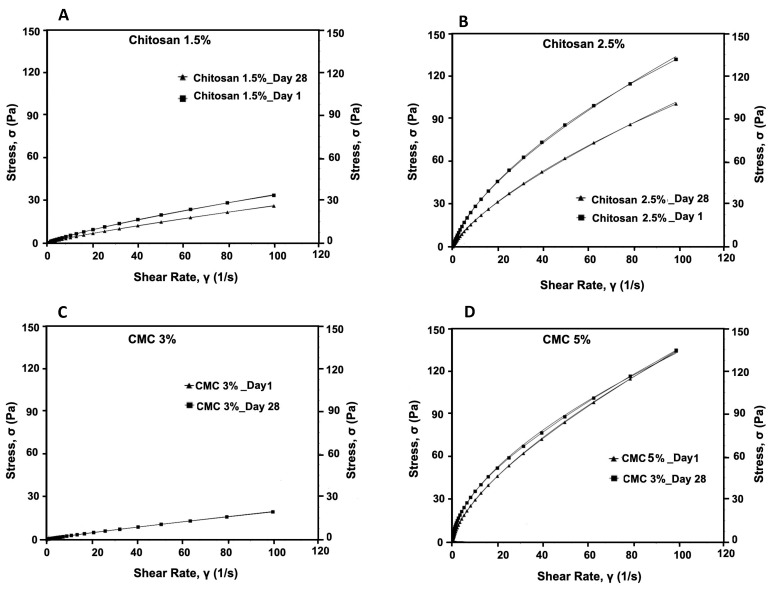
Flow curves of formulated hydrogels (**A**) 1.5% chitosan, (**B**) 2.5% chitosan, (**C**), 3% CMC, and (**D**) 5% CMC, measured on day 1 and day 28.

**Figure 5:**
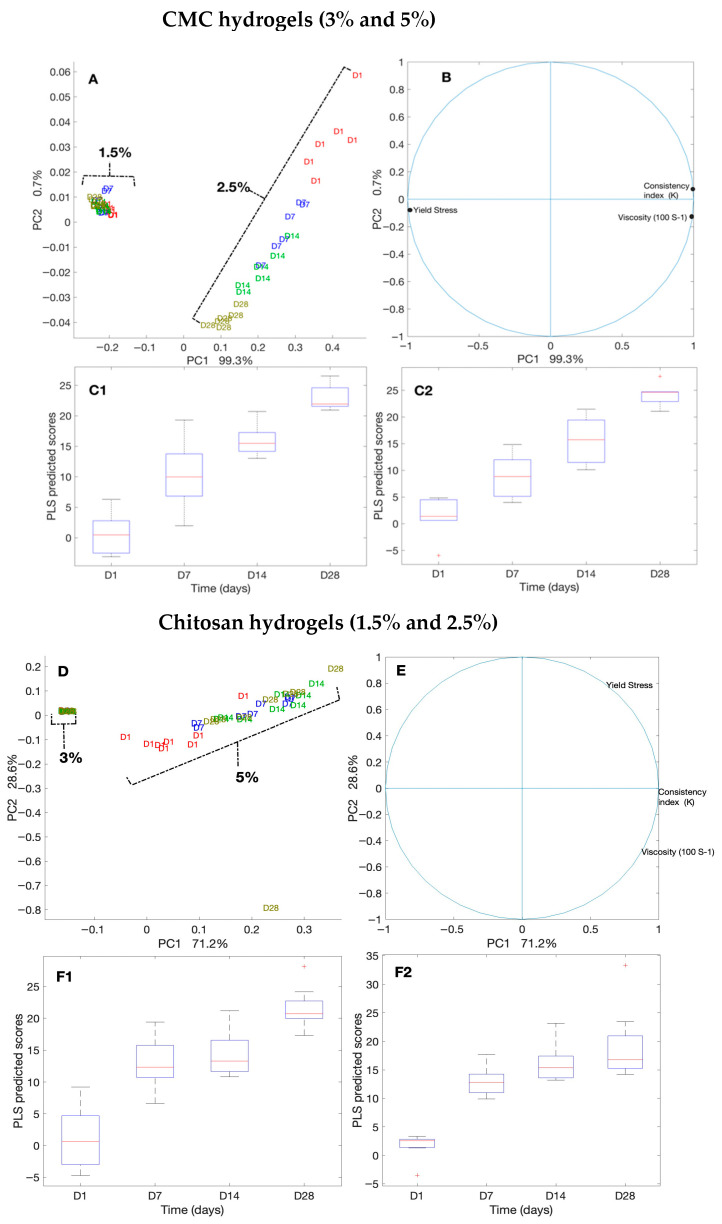
Principal component analysis (PCA) and partial least squares (PLS) regression of rheological measurements for CMC- and chitosan-based hydrogels over 28 days. (**A**–**C2**) CMC hydrogels (3% and 5%): (**A**) PCA score plots by polymer concentration and storage day; (**B**) PCA loading plots showing contributions of viscosity (100 s^−1^), consistency index (K), and yield stress; (**C1**,**C2**) PLS regression plots of PCA scores versus storage time. (**D**–**F2**) Chitosan hydrogels (1.5% and 2.5%): (**D**) PCA score plots, (**E**) PCA loading plots, and (**F1**,**F2**) PLS regression plots.

We evaluated the consistency and flow of the hydrogels using the Herschel–Bulkley model. The addition of antimicrobials at the final concentrations of 50 times that of the MIC to CMC and chitosan did not alter the flow and viscosity of the hydrogels compared with that of the controls. Thus, here, we present the results for our controls (3% and 5% CMC and 1.5% and 2.5% chitosan). While the viscosity of the CMC hydrogels at the shear rate of 100 (S^−1^), measured on day 1 and day 28, were unaltered (0.04 Pa.s and 0.26 Pa.s, respectively), that of chitosan decreased significantly (*p* < 0.001 (Table S1)) from 0.34 Pa.s to 0.25 Pa.s for 1.5% chitosan and from 1.32 to 0.98 for 2.5% chitosan. Moreover, we observed that the Herschel–Bulkley parameters were well fitted with R^2^ ≥ 0.9994 for all the CMC and chitosan hydrogels (Table 1 and Figure 4). All the hydrogels had *n* values that were lower than 1, indicating that they had pseudoplastic fluid characteristics. On the other hand, we observed a decrease in flow indices with an increase in polymer concentrations for both CMC and chitosan. Nevertheless, the effect that CMC concentration had on the *n* value was slightly greater than that of chitosan concentration. Notably, the rheological properties of the hydrogels did not vary over time; except for 2.5% chitosan hydrogel on day 28 as compared to day 1, showing a reduction in the consistency index. None of the samples exhibited yield stress. Using PCA and PLS, we further studied the correlations among hydrogels (Figure 5A,B). The plot of the PCA scores confirmed that, by increasing the concentration of polymers, viscosity (at 100/s), and consistency index, the K value increased. Furthermore, a PLS regression between the PCA scores and the shear time showed that the change in viscosity is less linear for the CMC-based gels than for chitosan, which indicates that rheological properties may not be constant over shearing time.

### 3.5. pH Evaluation

The pH values of the formulated hydrogel sanitizers are listed in Table 2. The results show that the formulated hydrogels were slightly acidic, with pH values ranging from 3.94 to 5.92. Specifically, the chitosan hydrogels (1.5% and 2.5%) had pH values of 3.96 and 4.87, respectively, which were more acidic than the CMC hydrogels (3% and 5%), which exhibited pH values of approximately 5.8 and 5.9, respectively. Notably, the addition of antimicrobials to the hydrogels did not significantly impact their pH compared to the controls, except for the addition of reuterin to the CMC hydrogels, which significantly reduced the pH by 0.7 units compared to the controls (*p* < 0.05). No significant changes in pH were observed during storage.

### 3.6. Antimicrobial Activity of Hydrogels

The MICs of the developed hydrogels on day 1 and post 28 days of storage at room temperature are summarized in Table 3. We noted that the polymer concentration did not have any impact on the antimicrobial activity of the formulated hydrogels. Remarkably, microcin J25 in CMC hydrogels exhibited the same level of antimicrobial activity against *S.* Newport ATCC 6962 as its pure solution. Microcin-J25-containing chitosan hydrogels exhibited similar antimicrobial activity as microcin J25 alone (MIC: 0.0356 µg/mL) against *S.* Newport ATCC 6962; however, a slight increase in MIC was observed in chitosan 2.5% (MIC 0.0712 µg/mL). The nisin Z solution showed a reduction in its antimicrobial activity during storage at room temperature, with MIC being increased eightfold after 28 days. A similar reduction in activity was observed in gel formulations. Specifically, nisin Z in chitosan hydrogels (1.5% and 2.5%) showed an eightfold increase in MIC during storage, while CMC hydrogels exhibited a more pronounced 16-fold increase in MIC, indicating a significantly weaker inhibition activity. On the other hand, pediocin PA-1 in CMC completely lost its antimicrobial activity during storage. However, pediocin PA-1 in chitosan hydrogels had a slightly lower antimicrobial activity against *L. monocytogenes* ATCC 19115 than pediocin PA-1 alone. Nonetheless, this activity remained unaltered during storage, whereas that of pediocin PA-1 alone was slightly reduced (MIC: 0.36 µg/mL). Reuterin in CMC hydrogels had an unaltered antimicrobial activity against *S.* Newport ATCC 6962. Moreover, these hydrogels retained their antimicrobial activity during the 28 days of storage period. However, CMC hydrogels containing pediocin PA-1 exhibited less antimicrobial activity (MIC: 11.2 µg/mL) than pediocin PA-1 alone (MIC: 0.36 µg/mL).

**Figure 6:**
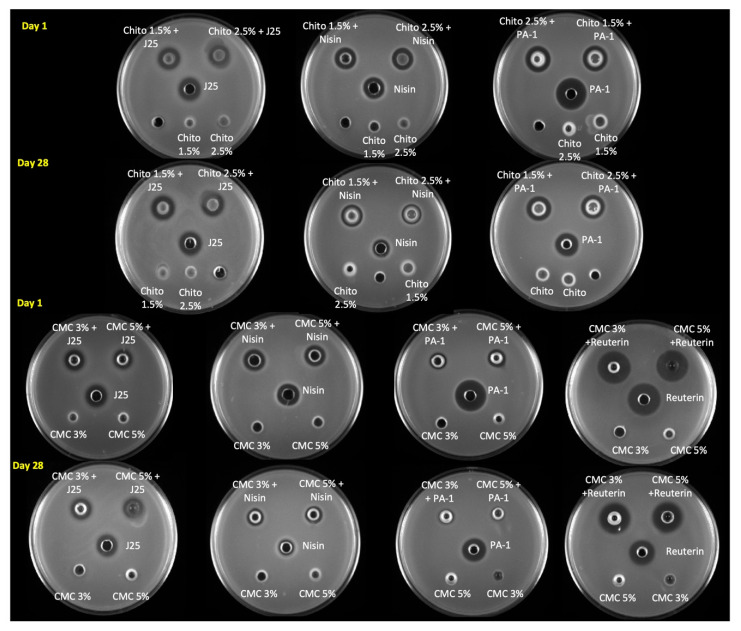
Antimicrobial activity of formulated hydrogels estimated using an agar well diffusion assay on day 1 and day 28. The antimicrobial activities of microcin J25, nisin Z, pediocin PA-1 and reuterin were tested against *S.* Newport ATCC 6962, *S. aureus* ATCC 6538, *L. monocytogenes* ATCC 19115 and *S.* Newport ATCC 6962, respectively. Control with hydrogels alone (without bacteriocins) were included on the same plate for comparison. Chito: Chitosan, J25: Microcin J25, PA-1: Pediocin.

The antibacterial activity of the different hydrogels was first assessed qualitatively using the agar well diffusion assay and by comparing the diameters of the inhibition (Figure 6). Microcin-J25-containing chitosan and CMC hydrogels had similar inhibition zones against *S.* Newport ATCC 6962 as pure microcin J25 (13 mm). Moreover, we did not observe any change in this regard during storage of the hydrogels. The activity of nisin Z against *S. aureus* ATCC 6538 decreased in both gel formulations (CMC and chitosan) as well as in solution form. The inhibition zone reduced from 15 mm to 13 mm in chitosan gels and from 15 mm to 12 mm in CMC gels. However, the inhibition zone of reuterin-containing hydrogels against *S.* Newport ATCC 6962 was measured to be the same as reuteirn alone (18 mm) at both 1 and 28 days of storage. Interestingly, pediocin PA-1 in the chitosan hydrogel had a slightly smaller inhibition zone (15 mm) than pediocin PA-1 alone (18 mm). While the inhibition zone remained unaltered for the hydrogel, that of the pediocin PA-1 solution decreased from 18 mm to 16 mm against *L. monocytogenes* ATCC 19115 after 28 days. Remarkably, the inhibition zone of pediocin PA-1 in CMC hydrogels was reduced to 10 mm on day 1 compared with pediocin PA-1, which remained at 18 mm even post storage; however, pediocin-PA-1-containing CMC hydrogels had no inhibition zone against *L. monocytogenes* ATCC 19115.

### 3.7. Sanitizing and/or Disinfectant Effect of Hydrogels as Determined by Log Reduction

**Figure 7:**
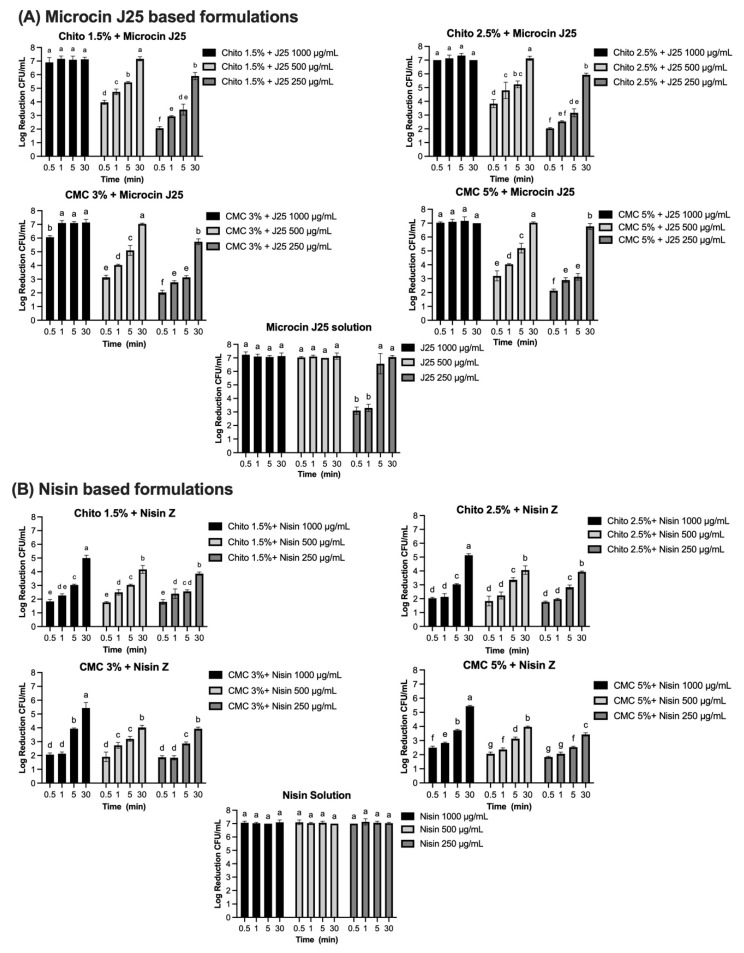

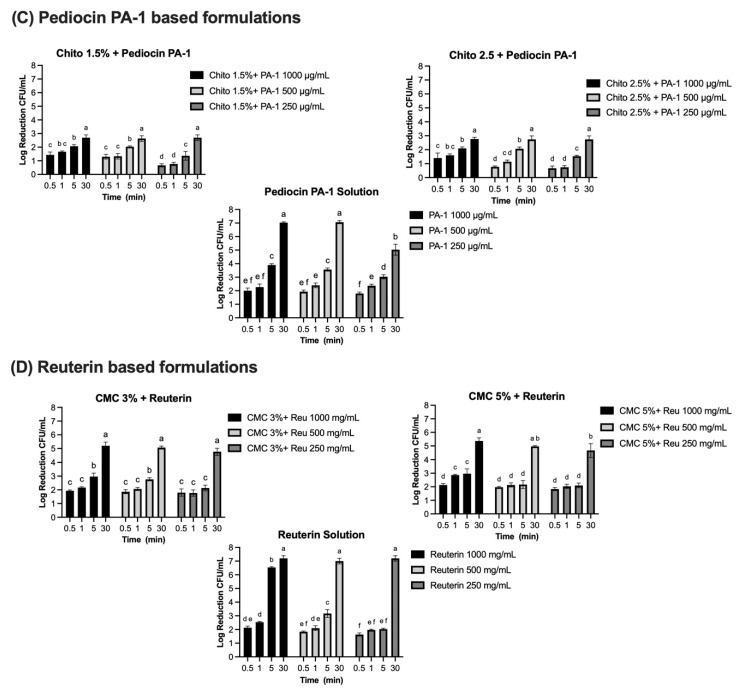
Log reduction (CFU/mL) of bacterial populations over time in solution and gel formulations containing microcin J25, nisin Z, pediocin PA-1, and reuterin. Panels: (**A**) Microcin J25 tested against *S.* Newport ATCC 6962, (**B**) nisin Z tested against *S. aureus* ATCC 6538, (**C**) pediocin PA-1 tested against *L. monocytogenes* ATCC 19115, and (**D**) reuterin tested against S. Newport ATCC 6962. Solutions and hydrogels (chitosan 1.5%, chitosan 2.5%, CMC 3%, and CMC 5%) were tested at three concentrations of each antimicrobial agent: 1000 µg/mL, 500 µg/mL, and 250 µg/mL for bacteriocins, and 1000 mg/mL, 500 mg/mL, and 250 mg/mL for reuterin. Time points included 0.5, 1, 5, 15, and 30 min. Bars represent mean values with error bars (standard deviations). Different superscript letters above the bars indicate statistically significant differences among treatments and time points based on two-way ANOVA followed by Tukey’s post hoc test. Treatments that share at least one letter are not significantly different (*p* ≥ 0.05), whereas treatments with different letters are significantly different (*p* < 0.05). Chito: chitosan, J25: Microcin J25, PA-1: pediocin PA-1.

The efficacy of bacteriocins and reuterin in both solution and gel formulations was evaluated against specific indicator strains using the AOAC protocol. Each compound was tested at varying concentrations and contact times, with results reported as log reductions (CFU/mL). The results for each antimicrobial, tested against its corresponding bacterial strain (*S.* Newport, *S. aureus*, or *L. monocytogenes*), are presented in Figure 7A–D, with significant reductions observed depending on the concentration, formulation, and contact time. Microcin J25 demonstrated potent antimicrobial activity against *S*. Newport ATCC 6962 (Figure 7A). In its solution form, a consistent 7-log reduction was observed across all contact times at 1000 µg/mL and 500 µg/mL (*p* > 0.05). At 250 µg/mL, 3.1 and 3.3 logs were obtained at 30 s and 1 min, respectively, reaching more than 5 logs by 5 min. When incorporated into 1.5% and 2.5% chitosan gels, microcin J25 maintained high activity. At 1000 µg/mL, a 7-log reduction was achieved across all contact times. At 500 µg/mL, initial reductions of 4 and 3.8 logs (in 1.5% and 2.5% gels, respectively) exceeded 5 logs after 5 min and reached 7 logs after 30 min. At 250 µg/mL, reductions ranged from 2 logs at 30 s to 5.9 logs at 30 min, with intermediate values of 3.4 and 3.2 logs at 5 min for 1.5% and 2.5% gels, respectively. Statistical analysis confirmed that both the chitosan concentration and contact time significantly influenced microbial reduction (*p* < 0.0001), with a smaller but significant interaction (*p* < 0.0001). In CMC-based gels (3% and 5%), microcin J25 exhibited similar trends. At 1000 µg/mL, a 7-log reduction was consistent across all contact times. At 500 µg/mL, reductions increased from 3.1–3.2 logs at 30 s to >5 logs at 5 min and 7 logs at 30 min. At 250 µg/mL, reductions ranged from 2.0–2.1 logs at 30 s to >5.7 logs at 30 min. The effects of CMC concentration and contact time were also significant (*p* < 0.0001), with a strong interaction between these factors (*p* < 0.0001).

Complete inhibition of *S. aureus* ATCC 6538 was observed with nisin Z in its solution form, achieving a consistent 7-log reduction across all tested concentrations (1000 µg/mL, 500 µg/mL, and 250 µg/mL) and contact times (Figure 7B). Statistical analysis confirmed no significant effects of concentration and contact time (*p* > 0.05) for the solution, indicating uniform antimicrobial activity under all conditions. When incorporated into gel formulations, the antimicrobial activity of nisin Z decreased compared to the solution form. In chitosan-based gels (1.5% and 2.5%), reductions ranged from 1.8–2 logs at 30 s to approximately 5 logs at 30 min at 1000 µg/mL. At 500 µg/mL, reductions ranged from 1.8 logs at 30 s to approximately 4.1–4 logs at 30 min, while, at 250 µg/mL, reductions were lower, ranging from 1.8 logs at 30 s to 3.9 logs at 30 min. Statistical analysis indicated significant effects of both concentration and contact time (*p* < 0.0001), with a smaller but significant interaction effect (*p* < 0.0001). Similarly, in CMC-based gels (3% and 5%), reductions followed a comparable pattern. At 1000 µg/mL, reductions ranged from 2.1–2.5 logs at 30 s to approximately 5.4 logs at 30 min. At 500 µg/mL, reductions ranged from 1.9–2.1 logs at 30 s to approximately 4 logs at 30 min. At 250 µg/mL, reductions were between 1.8 logs at 30 s and 3.4–4 logs at 30 min. The effects of concentration and contact time were statistically significant in CMC gels (*p* < 0.0001), though the interaction effect was smaller than in chitosan gels. Pediocin PA-1 exhibited significant antimicrobial activity against *L. monocytogenes* ATCC 19115, showing a clear dependence on concentration and contact time (Figure 7C). In its solution form, reductions of 2.0, 1.9, and 1.8 logs were observed at 1000 µg/mL, 500 µg/mL, and 250 µg/mL, respectively, within 30 s (*p* < 0.0001). By 5 min, reductions increased to 3.9, 3.6, and 3.0 logs, eventually exceeding 5 logs at 30 min for all concentrations. Complete 7-log reductions were achieved at the two highest concentrations. In chitosan-based gels (1.5% and 2.5%), the antimicrobial activity of pediocin PA-1 was reduced compared to its solution form, particularly at shorter contact times. At 1000 µg/mL, reductions reached 2.1 logs at 5 min and 2.7 logs at 30 min. At 500 µg/mL and 250 µg/mL, reductions remained below 3 logs for all contact times ≤5 min, achieving a maximum of 2.7 logs at 30 min.

Reuterin demonstrated concentration- and time-dependent antimicrobial activity against *S*. Newport ATCC 6962 (Figure 7D). In its solution form, significant reductions were observed at all tested concentrations. At 1000 mg/mL and 500 mg/mL, reductions of 2.1 and 1.9 logs, respectively, were achieved within 30 s, increasing to 6.5 and 3.2 logs at 5 min, and reaching a complete 7-log reduction at 30 min (*p* < 0.0001). At 250 mg/mL, reductions progressed from 1.6 logs at 30 s to 2 logs at 1 and 5 min, achieving 7 logs at 30 min (*p* < 0.0001). When incorporated into CMC-based gels (3% and 5%), the antimicrobial activity of reuterin was reduced compared to its solution form. At 1000 mg/mL and 500 mg/mL, reductions remained below 3 logs at contact times up to 5 min but exceeded 5 logs by 30 min. At 250 mg/mL, reductions were consistently below 3 logs for all contact times ≤5 min and reached a maximum of 4.7 logs at 30 min for both gel formulations. Statistical analysis revealed significant effects of concentration and contact time (*p* < 0.0001), with no significant interaction effect (*p* > 0.05).

## 4. Discussion

It is imperative to implement sanitation and disinfection programs to prevent bacterial contamination in the food, medical, and veterinary sectors. Over the last two decades, the use of ABHS and NABHS has substantially increased. However, concerns about the safety, environmental impact, and efficacy of these products persist [11]. Commercially available sanitizers may cause skin and eye irritation [37,38], and, in cases of misuse, they can even cause adverse health effects [39]. In addition, commercially available sanitizers may increase the risk of antimicrobial resistant in microorganisms, which is already a burden to public health [13]. Hayat et al. (2016) reported that 64% of *Pseudomonas aeruginosa* and 48% of *E. coli* strains were resistant to all commercially available sanitizers, moreover, all Gram-negative pathogens were resistant to Purel, Cool n Cool, Safegaurd, Freshup, and Insta foam sanitizers [40]. Thus, there is an urgent need to discover new, natural active compounds that can be used either in combination with or as replacements for commercial synthetic sanitizers. It is essential to develop natural formulations that are safe and have no negative impact on humans, animals, or the environment. Despite the urgent need for “green” sanitizers, only limited commercial options are currently available. Some products have been developed from natural antiseptic agents, such as plant extracts, essential oils, coconut oil, and extrudates [8]. Bacteriocins and reuterin, natural antimicrobials produced by bacteria, offer unique mechanisms of action and a lower risk of resistance development. In a previous study, we showed that bacteriocins, in combination with other antimicrobials (with different mechanisms of action), exert a synergistic antimicrobial effect and limit the risk of antimicrobial resistance against both of the compounds [41]. Notably, several studies investigating the sanitizing properties of bacteriocins have focused on their antibiofilm activity, particularly against pathogens relevant to healthcare and food-contact surfaces [42,43,44]. For instance, Kajwadkar et al. (2017) demonstrated that high-purity nisin, alone or in combination with sodium hypochlorite, effectively reduced both planktonic and biofilm populations of *Enterococcus faecalis* [45]. Kranjec et al. (2024) developed a bacteriocin-based coating strategy using enterocin EJ97-short and micrococcin P1 to prevent biofilm formation by vancomycin-resistant *E. faecium* on medical-device-related materials [43]. In addition, Javadi et al. (2025) reported that a bacteriocin derived from *Lactoplantibacillus plantarum* inhibited biofilm formation by multidrug-resistant *Acinetobacter baumannii*, a critical nosocomial pathogen [44]. Moreover, to date, the application of bacteriocins as sanitizers remains uninvestigated [46,47]. Therefore, one of our aims was to use bacteriocins and reuterin as active ingredients in combination with natural polymers to develop a safer and more natural hydrogel sanitizer. Bacteriocins and reuterin are water soluble and can be used in the liquid form as sprays. Potential slip hazards, application difficulty, excessive dripping, and low contact time make aqua sanitizers the least preferred sanitizers [48]. On the other hand, hydrogel preparations are becoming increasingly popular in the market due to their numerous advantages, including easy handling, no leakage, better practical application and contact time compared to liquid sanitizers, as well as the improved stability of active compounds. Hence, we developed hydrogels from the polysaccharide biopolymers CMC and chitosan. Both CMC and chitosan are recognized as safe and biocompatible polymers. The bacteriocins and reuterin used in this study were previously evaluated by our group for cytotoxicity, skin sensitization, and irritation, and showed no adverse effects at their effective antimicrobial concentrations [17]. However, the concentrations tested in this study (1000 µg/mL bacteriocins; up to 1000 mg/mL reuterin) are considerably higher than those previously assessed. Therefore, additional studies will be required to confirm safety at these concentrations for topical applications.

While CMC is an anionic water-soluble rheological modifier, it becomes insoluble when added to high concentrations of organic solvents. Previous studies have demonstrated the unsuitability of CMC in ABHSs, as it can only be used in gels containing 40% alcohol; this contradicts the WHO guidelines, which advise that hand sanitizers should contain >60% alcohol [49]. In the current study, we successfully developed CMC-based antimicrobial hydrogels using bacteriocins as active compounds and CaCl_2_ as a crosslinker.

Chitosan is a polycationic linear polysaccharide that is soluble in aqueous acidic solutions (pH < 6.5). Owing to its unique properties, such as biocompatibility, biodegradability, non-toxicity, and low allergenicity, chitosan has been used in various applications [50]. Chitosan has bio-adhesive properties and functions as a moisturizer by forming a film or coating that prevents moisture loss. This makes it advantageous for use in hand gels and on surfaces requiring longer contact times, offering a natural alternative to synthetic formulations [51,52]. Previously, chitosan has been used as a natural polymer to develop sanitizers; however, alcohol (60% by volume) remained the active ingredient [51].

Here, two concentrations of each polymer were selected to obtain varying viscosities for applications. Viscosity plays a key role in the functionality of sanitizer gels, varying based on the targeted application. Moreover, the intended use, performance, effectiveness and customer perception are closely related to the viscosity values of the products [53]. Greenaway et al. (2018) examined the compliance of different sanitizing formulations on a selected group of nurses, they found that all the nurses preferred the gel formulation to the liquid formulation because of application difficulties, unpleasant and uncontrolled dripping, and low coverage of the latter [48]. In contrast, gels with low viscosity and high spreadability are more appropriate for application on surfaces than those with higher viscosity. This is because gel spreadability is affected by viscosity, as low viscosity is associated with high spreadability and vice versa [54]. Thus, the more viscous formulations (5% CMC and 2.5% chitosan) are suitable as hand sanitizers, whereas the less viscous forms (CMC 3% and chitosan 1.5%) are suitable for application on surfaces. Although all the hydrogels exhibited pseudoplastic behavior, it was more prevalent at high concentrations than that at low concentrations, owing to increased molecular interactions. We also observed that increasing the concentration of CMC and chitosan decreased the tendency for shear thinning of the hydrogels (increase in flow behavior indices (*n* value) and decrease in K value). Notably, the tested concentrations of the antimicrobial compounds did not affect the flow behavior or consistency of the developed hydrogels. Moreover, during the 28-day storage period, the rheological properties of the hydrogels remained stable; nonetheless, a slight reduction in viscosity of chitosan formulations was also observed. This aligns with previous findings where viscosity changes in chitosan solutions were attributed to molecular interactions or structural adjustments influenced by acidic components in the medium [55]. These findings suggest that storage conditions and formulation properties may contribute to the slight decrease in viscosity observed in this study. Taken together, these results suggest that bacteriocins and reuterin can be used in these formulations without affecting the structure and functionality of the hydrogel.

It is essential to determine the pH of topically applied products in order to avoid skin irritation and inflammation; the ideal pH range is 4–7 [56,57]. The “normal” pH of the skin is acidic <5.5; this is necessary for dermal physiological processes such as skin barrier function, lipid synthesis and aggregation, as well as stratum corneum hemostasis [58]. In addition, pathogenic bacteria associated with skin infection can grow at a neutral pH, whereas the resident bacterial flora of the skin are preserved at an acidic pH [57]. In the current study, the formulated hydrogel sanitizers were slightly acidic. However, chitosan hydrogels were more acidic than CMC hydrogels because of the incorporation of 1% lactic acid in their formulation. Thus, slightly acidic hydrogel hand sanitizers have antimicrobial properties and are beneficial for dermal biological processes.

Chitosan is well-documented for its antimicrobial activity, which depends on factors such as its degree of acetylation (DA) and molecular weight (Mw). Higher DA is generally associated with reduced antimicrobial effectiveness, while the role of Mw varies across studies [59]. Some findings suggest that low Mw chitosan or oligomers are more effective against bacteria [60,61]. Additionally, some studies suggest that the relationship between MW and antimicrobial activity can vary depending on the bacterial species [62]. In this study, however, chitosan hydrogels without added bacteriocins or reuterin did not exhibit antimicrobial activity against the tested strains.

Microcin J25 demonstrated stability and activity in both chitosan and CMC hydrogels. In chitosan hydrogels, microcin J25 remained stable and active, likely due to the positively charged amine groups in chitosan, which do not interact adversely with the bacteriocin. This stability highlights the suitability of chitosan as a hydrogel matrix for microcin J25. Similarly, in CMC hydrogels, microcin J25 retained its antimicrobial properties. Its unique lasso peptide structure, where the N-terminal ring encircles eight residues and the C-terminal tail is tightly threaded [63], likely prevents the amine group of microcin J25 from binding to the carboxyl groups in CMC. This allows its release from the gel, increases its bioavailability, and maintains its inhibitory activity. This dual compatibility with both chitosan and CMC hydrogels makes microcin J25 a promising candidate for hydrogel-based sanitizers. Nisin Z, another bacteriocin, remained active in both CMC and chitosan hydrogels. However, its antimicrobial activity in solution decreased over time at room temperature, which may account for the reduced activity observed in the hydrogels after four weeks. Nisin Z is known to be unstable, and commercial preparations are often supplemented with stabilizers such as sodium chloride, sulfates, or surfactants to enhance their activity, though these additives can limit compatibility with some polymer systems [64]. Holcapkova et al. (2017) similarly reported a significant reduction in nisin Z activity after 55 days of storage at 25 °C, indicating the challenge associated with its stability [65]. Nisin Z stability is influenced by pH; as Delves-Broughton et al. (1996) reported, it is completely stable at pH 2.0 and at low temperatures (2–7 °C), while inactivation occurs above pH 7.0 at room temperature [66]. This likely explains why nisin Z in the more acidic chitosan hydrogels (pH 4.0–4.8) exhibited less activity loss compared to CMC hydrogels (pH 5.8–5.9). Optimizing pH and incorporating stabilizers could further enhance the stability and suitability of nisin Z for hydrogel-based sanitizers. Pediocin PA-1, which inhibits bacteria by forming pores in the cell membrane [67], showed reduced activity in CMC hydrogels, likely due to electrostatic complexation between its C-terminal amine and CMC carboxylates, which decreases peptide release/bioavailability [68]. However, in chitosan hydrogels, pediocin PA-1 remained stable and active due to the lack of adverse interactions with positively charged amine groups in chitosan. On day one, the MIC of pediocin PA-1 in the hydrogel was four times higher compared to the solution form, indicating a reduction in activity due to incorporation into the gel. However, over time, pediocin PA-1 activity in the solution decreased four times at room temperature, whereas its activity in the hydrogel remained consistent. This suggests that incorporating pediocin PA-1 into chitosan hydrogels helps maintain its antimicrobial efficacy over extended periods at room temperature. Reuterin, a low-molecular-weight antimicrobial compound, remained stable in CMC hydrogels during 28 days of storage. However, when incorporated into chitosan hydrogels, reuterin formed a solid gel and changed the color to red, likely due to a reaction between its carbonyl group and the amine group of chitosan. This behavior aligns with studies where aldehydes have been used as crosslinkers in chitosan-based hydrogels [69,70]. This interaction made the formulation unsuitable, leading to its exclusion from further analysis. Nevertheless, the stability of reuterin in CMC hydrogels, combined with its antimicrobial activity, makes it a strong candidate for use in hydrogel sanitizers. Overall, CMC hydrogels were ideal for use with reuterin, microcin J25, and nisin Z, whereas chitosan hydrogels provided a suitable matrix for microcin J25, nisin Z, and pediocin PA-1. Accordingly, biopolymer–bacteriocin interactions should be considered when designing hydrogel-based sanitizers.

The antimicrobial efficacy of microcin J25, nisin Z, pediocin PA-1, and reuterin was evaluated in both solution and gel formulations. Bacteriocins were tested at concentrations of 1000 µg/mL, 500 µg/mL, and 250 µg/mL, while reuterin was tested at 1000 mg/mL, 500 mg/mL, and 250 mg/mL to assess their suitability as sanitizers and disinfectants. These concentrations were selected to evaluate their bactericidal potential and determine their efficacy in achieving significant log reductions. Additionally, they were chosen to address potential challenges related to bioavailability and release in gel formulations, ensuring sufficient antimicrobial activity under practical application conditions. The AOAC 960.09 standard for food contact surface sanitizers [35], the FDA requirements for hand sanitizers [71], and the EPA criteria for non-food contact surface sanitizers (≥3-log reduction in 5 min) and disinfectants (≥6-log reduction in 10 min) [72] provide a comprehensive framework for evaluating antimicrobial efficacy. These standards emphasize both immediate microbial reduction and application-specific requirements, which are critical for assessing the antimicrobial activity of the hydrogels in this study. The results demonstrated that, while all tested compounds exhibited significant antimicrobial activity in solution, their efficacy in gel formulations was influenced by factors such as the concentration, contact time, and physicochemical properties of the gel matrix. Microcin J25 consistently achieved ≥7-log reductions at higher concentrations in solution, meeting or exceeding the stringent AOAC 960.09 benchmark for food contact sanitizers (≥5-log reduction within 30 s) [35]. However, at lower concentrations (250 µg/mL), its performance was time-dependent, achieving ≥6-log reductions after 5 min, aligning with the EPA criteria for disinfectants and non-food-contact surface sanitizers [72]. Since microcin J25 achieved ≥6-log reductions within 30 min at all tested concentrations, this suggests that, while its efficacy at lower concentrations is time dependent, it remains effective over prolonged exposure. When incorporated into chitosan and carboxymethyl cellulose (CMC) gels, microcin J25 retained its antimicrobial activity at 1000 µg/mL, achieving ≥7-log reductions within 30 s. At lower concentrations, it achieved ≥5-log reductions within 5 min in both gel matrices, meeting the EPA criterion for non-food contact surface sanitizers [72]. However, at lower concentrations and shorter contact times, log reductions were less pronounced, potentially due to reduced bioavailability, limited diffusion, or slower release rates in the gel matrix. By 30 min, all concentrations achieved ≥5-log reductions, suggesting that while microcin J25 in gel formulations may not provide immediate microbial inactivation, it remains effective in applications requiring prolonged contact.

Similarly, nisin Z exhibited strong bactericidal activity in solution, achieving complete inhibition of *S. aureus* ATCC 6538 across all concentrations and contact times, aligning with the stringent AOAC 960.09 benchmark for food contact sanitizers [35]. However, its efficacy in gel formulations was significantly reduced, with log reductions ranging from 1.8 to 5.4, depending on concentration and contact time. This decline, similar to microcin J25, likely results from diffusion limitations or interactions with gel components. While nisin Z meets regulatory standards in solution, further formulation optimization is required to enhance its gel-based performance.

Pediocin PA-1 exhibited limited bactericidal efficacy against *L. monocytogenes* ATCC 19115 at shorter contact times, with reductions of ≤2-log across all concentrations in solution after 30 s. However, after 5 min, all concentrations achieved ≥3-log reductions, meeting the EPA criteria for non-food-contact sanitizers but failing to reach the AOAC 960.09 requirement of a ≥5-log reduction for food contact surface sanitizers [35,72]. Incorporation into chitosan gels further diminished its efficacy, with maximum reductions of 2.7 logs across all concentrations after 30 min. This suggests that the bactericidal effect of pediocin PA-1 is dependent on sustained exposure, making it less suitable for applications requiring rapid microbial inactivation but potentially valuable for prolonged-contact applications. Reuterin demonstrated clear time- and concentration-dependent activity in solution, achieving 7-log reductions within 30 min. However, reductions at shorter contact times (30 s) were below thresholds required for food contact surface sanitizers as per AOAC 960.09 [35]. In CMC-based gels, reuterin retained substantial activity, achieving >5-log reductions at higher concentrations after 30 min. At lower concentrations, efficacy fell below all regulatory thresholds, suggesting that gel formulations may require prolonged surface retention to achieve sufficient microbial reductions.

Our results showed that reuterin and bacteriocins were more active in their solution forms compared to hydrogels; this difference is explained by diffusion barriers in the hydrogel matrix, which slow release and reduce immediate antimicrobial availability compared to freely soluble forms, with efficacy depending on both contact time and concentration. A key limitation associated with bacteriocins and reuterin is the time required to achieve meaningful bacterial reductions [47]. For example, Al-Seraih et al. (2017) demonstrated that a combination of enterocin B3A-B3B and nisin Z achieved only a 2-log reduction in *L. monocytogenes* biofilms on stainless steel after 24 h [73], reflecting the intrinsic tolerance of biofilms due to their extracellular polymeric matrix, which limits penetration of antimicrobials. Kajwadkar et al. (2017) further showed that high-purity nisin, alone or combined with sodium hypochlorite, significantly reduced both planktonic and biofilm populations of *E. faecalis* [45], indicating that synergy with chemical sanitizers may help overcome diffusion barriers. Singh et al. (2018) employed a gold nanocomposite incorporating pediocin AcH and *Listeria* adhesion protein, which specifically inhibited *L. monocytogenes* biofilms [74], highlighting that conjugation strategies can enhance bacteriocin targeting and retention on surfaces. Javadi et al. (2025) reported that a *Lpb. Plantarum*-derived bacteriocin suppressed biofilm formation by multidrug-resistant *Acinetobacter baumannii* [44]. Importantly, reuterin has been shown to inhibit biofilm formation by *Pseudomonas* spp. [75] and *Clostridium perfringens* [76], and to enhance biofilm eradication when combined with peracetic acid in multi-species biofilms [77], underlining the importance of both antimicrobial synergy and multi-target approaches for tackling resilient communities. Although the biofilm models used in these studies differ from the conditions of our work, collectively, they highlight both the time-dependent and context-dependent nature of bacteriocin and reuterin efficacy. In addition, our work focused on indicator strains such as *S. aureus* ATCC 6538, *S*. Newport ATCC 6962, and *L. monocytogenes* ATCC 19115, which are highly relevant to food safety applications; nevertheless, future studies should also include food industry isolates, and potentially hospital isolates, to further validate the performance of the hydrogels under real-world conditions. Incorporating antimicrobial agents into hydrogels often reduces their availability and immediate antibacterial efficacy due to diffusion barriers or slower release kinetics. For instance, vancomycin-loaded hydrogels demonstrated sustained release, achieving an 86% release rate by the third day, which, while beneficial for prolonged action, may not suffice for rapid microbial inactivation [62]. Similarly, hydrogel coatings have demonstrated diminished immediate efficacy attributed to slower agent diffusion and stability challenges [78]. These findings show the importance of optimizing gel formulations to balance rapid antimicrobial action with sustained release, particularly for applications requiring short contact durations.

Combining bacteriocins with other antimicrobials has been shown to enhance effectiveness. Bennett et al. (2022) reported that a nisin Z and reuterin consortium significantly outperformed individual bacteriocins, achieving up to 0.95- and 0.90-log reductions in *Staphylococci* and total bacterial counts, respectively, under practical conditions [79]. Phongphakdee et al. (2015) reported a 5-log reduction in *E. coli* and *Salmonella* on stainless steel within 15 min when nisin Z was combined with ethanol [80]. Although our study focused on bacteriocins alone, these findings suggest that synergistic agents or optimized release properties in hydrogel formulations could improve bacteriocin performance for rapid bacterial reductions in industrial applications.

While our study focused on pH stability, viscosity, and antimicrobial activity, other physicochemical parameters such as swelling index, mesh size, degree of crosslinking, and entrapment efficiency were not evaluated. These characteristics are important for predicting release behavior and long-term stability and should be addressed in future work to further optimize hydrogel formulations. Understanding the interactions between biopolymers and active ingredients based on their structure and functional groups will also be valuable for developing stable and effective hydrogel sanitizers. Optimizing these formulations is critical to enhance their potential for sanitation and disinfection, particularly in applications with strict time requirements.

## 5. Conclusions

This study is the first of its kind to successfully incorporate bacteriocins and reuterin as active ingredients in biopolymer (CMC and chitosan)-based hydrogel sanitizers. Our results revealed that the prediction of possible interactions between the functional groups of the selected bacteriocins, reuterin, and biopolymers can assist developing more efficient hydrogel formulations with higher activity and stability. Combining bacteriocins with different but complementary mechanisms of action can provide synergistic, broad-spectrum inhibition and reduce the risk of antimicrobial resistance development in bacteria. Validation of the efficacy of the developed hydrogel under real conditions, such as on surfaces or hands, is a prerequisite for its industrial-scale application. This includes testing using standard methods recognized by regulatory agencies to ensure compliance with safety and performance standards.

## Figures and Tables

**Table 1:** Herschel–Bulkley fitting parameters for CMC (3% and 5%) and chitosan (1.5% and 2.5%) hydrogels.

Formulas	Viscosity 100/s (Pa.s)	Yield Stress (Pa)	Flow Behavior Index (n)	Consistency Index (K) (Pa.s)	R^2^
3% CMC Day 1	0.04 ± 0.03	0.06	0.91 ± <0.01	0.06 ± <0.01	0.9999
3% CMC Day 28	0.04 ± <0.01	0.064	0.88 ± 0.01	0.07 ± 0.01	0.9999
5% CMC Day 1	0.28 ± 0.03	−1.317	0.66 ± 0.05	1.38 ± 0.38	0.9999
5% CMC Day 28	0.31 ± 0.03	−1.883	0.54 ± 0.03	2.52 ± 0.47	0.9994
1.5% Chitosan Day 1	0.33 ± 0.02	−0.1	0.82 ± <0.01	0.78 ± 0.06	0.9997
1.5% Chitosan Day 28	0.25 ± 0.01	0.472	0.87 ± <0.01	0.45 ± 0.03	0.9998
2.5% Chitosan Day 1	1.29 ± 0.07	0.1	0.66 ± 0.01	6.27± 0.53	0.9995
2.5% Chitosan Day 28	0.95 ± 0.05	0.605	0.74 ± 0.01	3.24 ± 0.32	0.9996

**Table 2:** The pH of the hydrogel formulations. The pH value is represented as the mean ± SD of two replicates (n = 2).

Formulation	pH
Chitosan1.5%	3.96 ± 0.1
Chitosan1.5% + microcin J25	3.95 ± 0.0
Chitosan1.5% + nisin Z	3.95 ± 0.1
Chitosan1.5% + pediocin PA-1	3.94 ± 0.1
Chitosan2.5%	4.87 ± 0.0
Chitosan 2.5% + microcin J25	4.85 ± 0.1
Chitosan 2.5% + nisin Z	4.88 ± 0.0
Chitosan2.5% + pediocin PA-1	4.75 ± 0.0
Chitosan1.5%	3.96 ± 0.1
Chitosan1.5% + microcin J25	3.95 ± 0.0
CMC 3%	5.8 ± 0.0
CMC3% + microcin J25	5.42 ± 0.0
CMC3% + nisin Z	5.81 ± 0.1
CMC3% + pediocin PA-1	5.76 ± 0.0
CMC3% + reuterin	5.1 ± 0.0
CMC 5%	5.92 ± 0.0
CMC5% + microcin J25	5.48 ± 0.0
CMC5% + nisin Z	5.74 ± 0.0
CMC5% + pediocin PA-1	5.63 ± 0.0
CMC5% + reuterin	5.2 ± 0.1

**Table 3:** Minimum inhibitory concentration of hydrogels and antimicrobial agents.

*S*. Newport ATCC 6962		
MIC		
Formulations	Day 1	Day 28
MccJ25	0.0356 µg/mL	0.0356 µg/mL
Chitosan 1.5% + MccJ25	0.0356 µg/mL	0.0356 µg/mL
Chitosan 2.5% + MccJ25	0.0356 µg/mL	0.0712 µg/mL
CMC 3% + MccJ25	0.0356 µg/mL	0.0356 µg/mL
CMC 5% + MccJ25	0.0356 µg/mL	0.0356 µg/mL
Reuterin	0.25 mg/mL	0.25 mg/mL
CMC 3% + reuterin	0.25 mg/mL	0.25 mg/mL
CMC 5% + reuterin	0.25 mg/mL	0.25 mg/mL
***L. monocytogenes* ATCC 19115**		
**MIC**		
	**Day 1**	**Day 28**
Pediocin PA-1	0.36 µg/mL	1.44 µg/mL
Chitosan 1.5% + pediocin PA-1	1.44 µg/mL	1.44 µg/mL
Chitosan 2.5% + pediocin PA-1	1.44 µg/mL	1.44 µg/mL
CMC 3% + pediocin PA-1	11.2 µg/mL	>102.4 µg/mL
CM 5% + pediocin PA-1	11.2 µg/mL	>102.4 µg/mL
***S. aureus* ATCC 6538**		
Nisin Z	2 µg/mL	16 µg/mL
Chitosan 1.5% + nisin Z	2 µg/mL	16 µg/mL
Chitosan 2.5% + nisin Z	2 µg/mL	16 µg/mL
CMC 3% + nisin Z	2 µg/mL	32 µg/mL
CMC 5% + nisin Z	2 µg/mL	32 µg/mL

## Data Availability

The raw data supporting the conclusions of this article will be made available by the authors on request.

## Supplementary Materials

The following supporting information can be downloaded at: https://www.mdpi.com/article/10.3390/microorganisms13102249/s1, Table S1: Statistical analysis (Tukey’s *t* test) of viscosity (100 S-1) and flow indices for chitosan and CMC hydrogels at day 1 and day 28 for at least three replicates.
