# Supplementary material for: Stability and Antimicrobial Efficacy of Reuterin and Bacteriocins (Microcin J25, Nisin Z, and Pediocin PA-1) in Chitosan- and Carboxymethyl-Cellulose-Based Hydrogels
Source: Microorganisms. 2025 Sep 25;13(10):2249. doi: 10.3390/microorganisms13102249 (PMC12566303; doi:10.3390/microorganisms13102249)
Supplement: Supplementary file 1 [file microorganisms-13-02249-s001.zip › microorganisms-3794589-supplementary.pdf]

Table 1. Statistical analysis *(Tukey's t test)* of viscosity (100 S^{-1}) and flow indices for chitosan and CMC hydrogels at day 1 and day 28 for at least three replicates.

**Viscosity 100 S-1**		
Tukey's multiple comparisons test	Mean Diff.	Significant?
Chitosan 1.5%_Day 1 vs. Week 4	0.06557	Yes **
Chitosan 2.5%_Day 1 vs. Week 4	0.3154	Yes ***
CMC 3%_Day 1 vs. Week 4	0.0006777	No
CMC 5%_Day 1 vs. Week 4	-0.02078	No
**Consistency index (K value)**		
Tukey's multiple comparisons test	Mean Diff.	Significant?
Chitosan 1.5%_Day 1 vs. Week 4	0.3295	No
Chitosan 2.5%_Day 1 vs. Week 4	3.028	Yes ***
CMC 3%_Day 1 vs. Week 4	-0.006360	No
CMC 5%_Day 1 vs. Week 4	-1.144	Yes ***
**Flow behavior (n value)**		
Tukey's multiple comparisons test	Mean Diff.	Significant?
Chitosan 1.5%_Day 1 vs. Week 4	-0.05657	Yes ***
Chitosan 2.5%_Day 1 vs. Week 4	-0.07605	Yes ***
CMC 3%_Day 1 vs. Week 4	0.02488	No
CMC 5%_Day 1 vs. Week 4	0.1199	Yes ***
